# Development and Validation of a Novel Triage Tool for Predicting Cardiac Arrest in the Emergency Department

**DOI:** 10.5811/westjem.2021.8.53063

**Published:** 2022-02-23

**Authors:** Chu-Lin Tsai, Tsung-Chien Lu, Cheng-Chung Fang, Chih-Hung Wang, Jia-You Lin, Wen-Jone Chen, Chien-Hua Huang

**Affiliations:** * National Taiwan University Hospital Department of Emergency Medicine, Taipei, Taiwan; † National Taiwan University College of Medicine, Department of Emergency Medicine, Taipei, Taiwan

## Abstract

**Background:**

Early recognition and prevention of in-hospital cardiac arrest (IHCA) have played an increasingly important role in the chain of survival. However, clinical tools for predicting IHCA are scarce, particularly in the emergency department (ED). We sought to estimate the incidence of ED-based IHCA and to develop and validate a novel triage tool, the Emergency Department In-hospital Cardiac Arrest Score (EDICAS), for predicting ED-based IHCA.

**Methods:**

In this retrospective cohort study we used electronic clinical warehouse data from a tertiary medical center with approximately 100,000 ED visits per year. We extracted data from 733,398 ED visits over a seven-year period. We selected one ED visit per person and excluded out-of-hospital cardiac arrest or children. Patient demographics and computerized triage information were included as potential predictors.

**Results:**

A total of 325,502 adult ED patients were included. Of these patients, 623 (0.2%) developed ED-based IHCA. The EDICAS, which includes age and arrival mode and categorizes vital signs with simple cut-offs, showed excellent discrimination (area under the receiver operating characteristic [AUROC] curve, 0.87) and maintained its discriminatory ability (AUROC, 0.86) in cross-validation. Previously developed early warning scores showed lower AUROC (0.77 for the Modified Early Warning Score and 0.83 for the National Early Warning Score) when applied to our ED population.

**Conclusion:**

In-hospital cardiac arrest in the ED is relatively uncommon. We developed and internally validated a novel tool for predicting imminent IHCA in the ED. Future studies are warranted to determine whether this tool could gain lead time to identify high-risk patients and potentially reduce ED-based IHCA.

## INTRODUCTION

In-hospital cardiac arrest (IHCA) has increasingly been recognized as a separate entity from out-of-hospital cardiac arrest (OHCA).^1^ Out-of-hospital cardiac arrests are typically sudden events that have a primary cardiac cause, whereas IHCAs occur typically in older patients with both cardiac and respiratory causes.^1^ Although IHCA has been traditionally understudied, recent studies have begun to reveal its incidence and survival using data from large clinical registries, such as the United Kingdom National Cardiac Arrest Audit (UK NCAA) database^2^ and the American Heart Association’s (AHA) Get With the Guidelines-Resuscitation registry.^3^ In the United States, the incidence of adult-treated IHCA was about 10 per 1000 bed-days (~290,000 patients per year), about 10% of which occurred in the emergency department (ED).^4^,^5^

There has been increasing interest in research on ED-based IHCA.^6^ Patients in the ED may be more prone to IHCA because of infrequent physiologic measurements, ED crowding, and unstable patient conditions.^7^ However, previous IHCA studies have focused primarily on ward patients,^8^,^9^ with few studies attempting to validate ward-based IHCA prediction tools in selected ED patients.^10^–^12^ To our knowledge, two ED-based risk prediction tools have been developed; however, they were used to predict in-hospital mortality instead of imminent cardiac arrest in the ED.^13^,^14^ Emergency department-based IHCA events requiring resuscitation are rarer and more difficult to predict than the downstream endpoint of mortality (with or without resuscitation), but are highly relevant to patients and clinicians. Taken together, as EDs around the world see more and sicker patients, there is a need to understand the incidence of IHCA in the ED and to develop better tools at triage to predict catastrophic IHCA events in a crowded ED.

In this study, we aimed to estimate the incidence of cardiac arrest in the ED and to develop and validate a novel triage tool for predicting IHCA in the ED.

## METHODS

### Study Design and Setting

We conducted a retrospective cohort study using data from the integrated Medical Database of National Taiwan University Hospital (NTUH). This database serves as a central clinical data warehouse for all electronic health records (EHR) in the healthcare system (a main hospital and six branch hospitals), including inpatient, outpatient, and ED records. The electronic database houses a variety of information, including demographics, diagnosis, treatment, imaging, laboratory, prescription, nursing, billing, and administrative data. The database is maintained and updated by dedicated research personnel and has been used for previous research studies.^15^,^16^

For the current study, we retrieved seven years of ED data from the main hospital between January 1, 2009 and December 31, 2015. The NTUH main hospital is a tertiary academic medical center with approximately 2400 beds and 100,000 ED visits per year. The ED also manages an ED observation unit (EDOU), which is staffed by emergency physicians. This study was approved by the NTUH Institutional Review Board, which waived the requirement for patient informed consent.

### Study Population

We extracted data from 733,398 ED visits over the seven-year period, including those in the EDOU. For repeat visits, we selected the last visit per patient to maximize statistical power for cardiac arrest analysis. Because cardiac arrest may result in death during an ED visit that became the last visit for the patient. We further excluded patients aged <18 years or those who presented with OHCA. The OHCA population was identified by the structured chief complaint list in the computerized triage system. Few OHCA patients may have a return of spontaneous circulation prior to ED arrival. These patients were still excluded from our study, as we focused on the IHCA population. The subject selection process is shown in Supplementary eFigure 1.

**Population Health Research Capsule**

What do we already know about this issue?

*Early recognition of in-hospital cardiac arrest (IHCA) is important in the chain of survival; however, clinical tools for predicting IHCA in the ED are scarce.*

What was the research question?

*We sought to develop and validate a novel triage tool for predicting ED-based IHCA.*

What was the major finding of the study?

*The Emergency Department In-hospital Cardiac Arrest Score (EDICAS) was developed and internally validated for predicting imminent IHCA in the ED.*

How does this improve population health?

*Future studies are warranted to determine whether this novel tool could potentially reduce ED-based IHCA.*

### Variables

We extracted patient demographics and the following time-stamped clinical information at triage: chief complaint on presentation; mode of arrival; transfer status; vital signs (temperature, heart rate, systolic and diastolic blood pressure, respiratory rate, oxygen saturation); and levels of consciousness coded using the Glasgow Coma Scale (GCS). The data extractors were hospital information technology engineers who were blinded to the study hypothesis. After investigator meetings, data underwent rigorous electronic cleaning, and invalid data were set to missing values (eg, out-of-range vital signs). For example, we defined that the respiratory rate ranged between 0–50 breaths per minute.

At ED triage, when assessing levels of consciousness the triage nurse also indicated whether there was an acute change in levels of consciousness from baseline on the structured EHR. Pain scores were evaluated on a numeric rating scale (NRS) of 0 to 10, with 0 being no pain and 10 being the worst pain imaginable. We further categorized the NRS scores into no (0), mild (1–3), moderate (4–6), and severe (7–10) pain.^17^ We also classified levels of consciousness as severe coma (GCS ≤ 8), moderate coma (9–12), and minor coma to normal status (GCS ≥ 13).^18^ Patients with special conditions, such as aphasia, tracheostomy, and endotracheal tube intubation, were classified as “other” on the GCS evaluation. We classified ED as day (7 am–2:59 pm), evening (3 pm–10:59 pm), and night (11 pm–06:59 am) shifts. The primary diagnosis fields of ED discharge codes were grouped into clinically meaningful categories using the Clinical Classification Software for the *International Classification of Diseases*, *9^th^ Revision, Clinical Modification*.^19^

We extracted the five-level computerized Taiwan Triage and Acuity Scale (TTAS), which contains information on 179 structured chief complaints. Based on computerized algorithms, the TTAS classifies patients in the following order of acuity: level 1, resuscitation; level 2, emergent; level 3, urgent; level 4, less urgent; and level 5, non-urgent. The TTAS was adapted from the Canadian Triage and Acuity Scale and has been validated against hospitalization, length of stay in the ED, and resource utilization.^20^

### Outcome Measure

We identified the primary outcome measure, ED-based IHCA, via a cardiopulmonary resuscitation (CPR) code (ie, treated cardiac arrest). Patients with do-not-resuscitate (DNR) status were not counted as treated cardiac arrests. Per the consensus guidelines on reporting IHCA,^1^ we calculated the ED-based IHCA incidence as the number of treated arrests (numerator) divided by the ED study population (denominator). The secondary outcome was mortality in the ED.

### Statistical Analysis

Summary statistics are presented as proportions (with 95% confidence intervals [CI]), means (with standard deviations), or medians (with interquartile ranges). We examined bivariate associations using Student’s t-tests, Mann-Whitney tests, chi-square tests, and chi-square trend tests, as appropriate. We used complete-case analysis, as the vast majority of variables in the analysis had few or no missing values except for respiratory rate. We used multivariable logistic regression to examine the independent factors associated with ED-based IHCA. Variables associated with the primary outcome measure at *P* <0.10 in bivariate analyses were considered for inclusion in the multivariable analysis. To determine the functional form and cut-off points used for continuous predictors, we grouped these predictors into bins of equal width to see whether log odds of ED-based IHCA changed at certain inflection points. Inflection points were also chosen based on inspection of locally weighted least squares regression smoother. After constructing a full multivariable model, we selected a parsimonious model using the least absolute shrinkage and selection operator. This operator uses a shrinkage parameter to perform the variable selection by penalizing the coefficients of less strong predictors, thereby mitigating potential model overfitting.

We used the variables and their odds ratios (OR) in the condensed model to derive an ED In-hospital Cardiac Arrest Score (EDICAS). The eight-item composite score ranges from 0–13, in which GCS and acute change in consciousness may be used interchangeably. Sensitivity and specificity were calculated with varying cut-off points. We evaluated the discriminatory ability of the final models by using the area under the receiver operating curve (AUROC). The CI of the AUROC was calculated using the DeLong method.^21^ We re-evaluated the performance of the final model by 10-fold cross-validation to assess potential model overfitting, and the average AUROC was reported.^22^ We also computed model AUROCs by using other early warning scores (EWS), including the National Early Warning Score (NEWS)^8^, ^23^ and Modified Early Warning Score (MEWS)^9^ for comparison purposes. Finally, a net reclassification improvement was calculated to estimate the benefit of the EDICAS as compared to the TTAS triage levels.

All OR and beta-coefficients are presented with 95% CIs. We performed all analyses using Stata 16.0 software (StataCorp, College Station, TX). All *P*-values are two-sided, with *P* <0.05 considered statistically significant.

## RESULTS

Of 733,398 ED visits during the seven-year study period, 405,891 unique patient visits were included. After excluding children aged <18 years or patients with OHCA, we included 325,502 patient visits in the analysis. The patient selection process is shown in Supplementary eFigure 1. Overall, the mean age of these patients was 49 years, and 53% were women. The overall incidence of ED-based IHCA was 0.19% (95% CI: 0.18%–0.21%). As shown in Table 1, compared with non-IHCA patients, patients with IHCA were much older and predominantly male. In terms of season, weekend, or time of ED presentation, there were no significant differences between the two groups. Compared with non-IHCA patients, IHCA patients were more likely to arrive by ambulance, to be transferred from other facilities, and to present with dyspnea and chest pain. Patients with IHCA were also more likely to present with higher triage levels, with impaired consciousness or acute change in consciousness, but were less likely to express pain of any levels. Regarding triage vital signs, IHCA patients presented with higher heart and respiratory rates but lower oxygen saturation and systolic blood pressure. In the IHCA group, the median time to CPR was about seven hours. The median length of ED stay was about nine hours in the IHCA group and about three hours in the non-IHCA group. The admission and ED mortality rates were high among patients with IHCA. The most common discharge diagnoses/symptoms for ED patients with IHCA were pneumonia, chest pain, and gastrointestinal hemorrhage (Supplementary eTable 1).

Multivariable analysis showed that factors associated with an increased risk of ED-based IHCA included older age, arrival by ambulance, transfers, day and night (vs evening) shifts, low systolic blood pressure (<90 millimeters of mercury [mm Hg]), brady- (<60/minute) and tachycardia (>90/minute), low oxygen saturation (<95%), tachypnea (≥22/min), hypothermia (<36°C), and triage levels 1 and 2 (Table 2). By contrast, moderate and severe pain (vs no pain) and triage levels 4 and 5 were associated with a decreased risk of IHCA in the ED.

**Figure 1:**
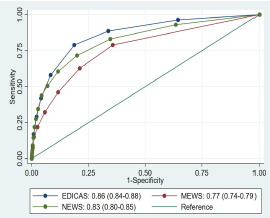
Receiver operating characteristic curves for three early warning scores: EDICAS, MEWS, and NEWS.* The numbers in parentheses indicate the confidence intervals for the area under the receiver operating characteristic curve. The diagonal line represents a model of no discriminatory ability. *EDICAS, Emergency Department In-hospital Cardiac Arrest Score; *MEWS*, Modified Early Warning Score; *NEWS*, National Early Warning Score.

A condensed model of multivariable analysis included the following strong predictors of ED-based IHCA: age ≥ 65years; arrival by ambulance; low systolic blood pressure (<90 mm Hg); brady- (<60/minute [min]) and tachycardia (>90/min); low oxygen saturation (<95%); tachypnea (≥22/min); hypothermia (<36°C); and GCS<15 (Table 3). This condensed model showed excellent discrimination (AUROC, 0.87; Supplementary eFigure 2) and maintained its discriminatory ability (AUROC, 0.86) in 10-fold cross-validation. Previously developed early warning scores showed lower AUROC (0.77 for MEWS and 0.83 for NEWS, *P* <0.001 for either one vs EDICAS) when applied to our ED population (Figure 1).

Based on the condensed model, we developed a predictive tool, the EDICAS (Table 4). The eight-item composite score ranges from 0–13, in which GCS and acute change in consciousness may be used interchangeably. The alternative EDICAS model with acute change in levels of consciousness yielded similar results (Supplementary eTable 2). We defined an EDICAS of 0–2, 3–5, 6+ as low-, medium-, and high-risk categories, respectively. Most patients were in the low-risk group (81%), and others were in the medium-risk (17%) and high-risk groups (2%). An EDICAS of 6+ corresponded to a specificity of 98%, and a positive likelihood ratio of 12.7 (Table 5). Compared with the TTAS triage levels, the EDICAS risk categories yielded a net reclassification improvement of 19%. In the IHCA group, 14% were correctly reclassified using the EDICAS. Finally, the EDICAS also showed outstanding discrimination power in predicting ED mortality (AUROC, 0.91).

## DISCUSSION

In this ED-based study of 325,502 patients, we found that a relatively small fraction of patients (2 in 1000) developed IHCA. A novel and simple eight-item triage score predicted imminent ED-based IHCA with excellent discriminatory power, with an AUROC outperforming previous early warning scores. Future prospective studies are warranted to replicate our results and to determine whether the implementation of this tool could actually gain lead time to identify high-risk patients and potentially reduce devastating, ED-based IHCA events.

Despite the catastrophic nature of IHCA, the epidemiology of IHCA remains largely unknown worldwide.^24^ The vast majority of data came from the AHA and UK NCAA databases.^2^,^4^ The most recent US data reported an estimated incidence of IHCA of 9–10 per 1000 admissions,^4^ while the UK database provided a much smaller figure of IHCA of 1.6 per 1000 admissions.^2^ Both data sources suggested that approximately 10% of IHCA events occurred in the ED. However, the denominators of the abovementioned IHCA incidence rates were based on inpatient admissions, not ED visits. Given a larger denominator of ED visits and a subset of IHCA events occurring in the ED, the ED-based IHCA incidence was expected to be lower than the inpatient IHCA incidence. Indeed, our estimate of IHCA incidence was about 1.9 per 1000 ED visits, which was much lower than the US inpatient IHCA incidence. This figure may be useful for benchmarking future ED-based IHCA studies.

Regarding seasonal variation, the incidence of ED-based IHCA in our study peaked in the winter, paralleling that in the UK study.^2^ The increased ED-based IHCA events during the winter months may result from concurrent increased cardiovascular and respiratory diseases. Interestingly, the EDICAS also peaked in the winter (mean score, 1.47 in the winter vs 1.26 in the summer [data not shown]), supporting its concurrent validity. In terms of disease burden, with approximately seven million ED visits annually in Taiwan,^25^ this small incidence could potentially translate into ~14,000 IHCA events in the ED. Given a high mortality rate of ~80% for IHCA patients,^2^,^24^,^26^ many patients could benefit from early recognition of IHCA.

As shown in recent resuscitation guidelines,^27^ the first link of the in-hospital chain of survival is early recognition and prevention of IHCA. Emergency department-based IHCA has increasingly been recognized as a distinct entity from IHCAs in other locations, such as on the ward or in the intensive care unit.^24^,^28^ The median time to cardiac arrest was about two days in previous reports of ward patients,^5^,^29^ while ours was about seven hours. As our ED also manages an EDOU, some of the IHCA patients deteriorated later in their ED course, which might have lengthened the time to arrest. Nonetheless, the relatively shorter time to cardiac arrest in ED patients suggests the time-sensitive nature of some emergencies, such as acute respiratory compromise and acute coronary syndrome. Indeed, the most common presentations were dyspnea and chest pain in our IHCA population, with discharge codes suggesting possible diagnoses of pneumonia, shock, and syncope. Despite the shorter time to cardiac arrest, ED-based IHCAs have been linked to improved survival to hospital discharge than those occurring in other locations, probably due to 24-hour on-site physician coverage and quick access to advanced life support equipment.^5^ With these advantages, early recognition of imminent IHCA in the ED should have great potential to reverse the course of further deterioration.

We developed and validated an ED-specific, eight-item EWS that we call the EDICAS, which is intended to be used at ED triage to augment traditional triage in predicting imminent cardiac arrest. After a stringent selection of the strongest predictors, the condensed model comprised older age, arrival mode, and primarily vital signs. Some of the original predictors, such as time of presentation and pain score, were selected out due to concerns of model overfitting. The inclusion of older age in the EDICAS highlights the importance of age in the triage process^30^ because a previous study suggested that older patients requiring an immediate life-saving intervention were more likely to be missed by using the Emergency Severity Index at triage.^31^ The addition of arrival by ambulance to this ED-specific tool seems quite reasonable because this variable should be readily available in most EDs. Some of the cut-offs for vital signs in the EDICAS were much simpler than those in previous EWS,^8^,^9^,^13^ making it easier to calculate and use at ED triage or before seeing the patient. We speculate that some unique characteristics of the ED population, such as a broad spectrum of acuity, may have contributed to a sharp contrast in severity between urgent and critically ill patients, resulting in fewer vital-sign cut-offs in the EDICAS. For example, the EDICAS does not assign points for high body temperature and high blood pressure as other EWSs do. Along these lines, previous ED-based studies showed that hyperthermia and high blood pressure did not seem to be strongly associated with adverse events in the ED.^32^,^33^

We defined an EDICAS of 3–5 as a medium-risk category, which may be used to flag patients needing an urgent physician assessment, particularly those who are initially triaged to lower levels. We also defined an EDICAS of 6 or above as a high-risk category as it corresponded to a specificity of 98% and a positive likelihood ratio of 12.7, both of which could raise the probability of finding rare ED-based IHCA. Similar to the recommendations from the NEWS Working Group,^34^ we recommend that a high-risk EDICAS at triage should prompt emergency assessment by an attending physician in the ED and/or transfer of the patient to a critical care area, if available. Physicians’ bedside reassessment is important to further increase the positive predictive value of IHCA, ie, confirming imminent IHCA after using the EDICAS as a screening measure. Furthermore, a continuous assessment of patient status would be prudent, as a previous ED study found an increase in NEWS after ED management and the use of a vasoactive agent predicted ED-based IHCA.^35^

## LIMITATIONS

This study has some potential limitations. First, this was a single-center study at a tertiary medical center, and our findings may not be generalizable to hospitals of different settings. Second, we did not externally validate our prediction model, and further studies are needed to evaluate our model performance in different patient populations. Third, our predictive tool is intended to be used at ED triage; whether a continuous assessment of EDICAS would also be predictive of ED-based IHCA requires further research.

## CONCLUSION

In this large study of 325,502 adult ED patients, 0.2% developed IHCA. We developed and validated a novel eight-item ED triage tool for predicting imminent IHCA in the ED with excellent discriminatory ability. While promising, our results need to be replicated in other EDs. Further research is also warranted to test whether this tool could gain lead time to identify high-risk patients and potentially reduce ED-based IHCA and associated deaths.

## Supplementary Information







## Figures and Tables

**Table 1:** Baseline clinical characteristics of emergency department patients by in-hospital cardiac arrest status.

Variable	IHCA (N = 623)	No IHCA (N = 324,879)	*P*-value
Age, mean (SD), yr	67.1 (16.5)	48.6 (19.9)	<0.001
Female gender, N (%)	241 (38.7)	172,109 (53.0)	<0.001
Season, N (%)			0.338
Spring (Mar. – May)	163 (26.2)	83,330 (25.6)	
Summer (June – Aug.)	148 (23.8)	81,779 (25.2)	
Fall (Sep. – Nov.)	139 (22.3)	78,565 (24.2)	
Winter (Dec. – Feb.)	173 (27.8)	81,205 (25.0)	
Weekend, N (%)	183 (29.4)	102,959 (31.7)	0.214
Time of presentation, N (%)			0.069
7 AM to 2:59 PM	271 (43.5)	127,477 (39.2)	
3 PM to 10:59 PM	236 (37.9)	136,297 (42.0)	
11 PM to 6:59 AM	116 (18.6)	61,105 (18.8)	
Arrival by ambulance, N (%)	245 (39.3)	30,453 (9.4)	<0.001
Transfer in, N (%)	104 (16.7)	23,008 (7.1)	<0.001
Presenting chief complaint, N (%)			<0.001
Abdominal pain	33 (5.3)	38,480 (11.9)	
Fever	41 (6.6)	23,198 (7.1)	
Dyspnea	163 (26.2)	16,639 (5.1)	
Dizziness	15 (2.4)	14,830 (4.6)	
Chest pain	41 (6.6)	9,951 (3.0)	
Other	328 (52.8)	219,994 (68.1)	
Triage level, N (%)			<0.001
1	254 (40.8)	8,519 (2.6)	
2	226 (36.3)	82,112 (25.3)	
3	135 (21.7)	191,290 (58.9)	
4	6 (1.0)	30,938 (9.5)	
5	2 (0.3)	12,020 (3.7)	
Pain score, N (%)			<0.001
Severe (7–10)	62 (10.4)	71,071 (22.0)	
Moderate (4–6)	40 (6.7)	67,971 (21.0)	
Mild (1–3)	5 (0.8)	13,410 (4.2)	
No pain (0)	488 (82.0)	170,527 (52.8)	
GCS, N (%)			<0.001
13–15	461 (74.0)	315,070 (97.0)	
9–12	54 (8.7)	4,663 (1.4)	
3–8	75 (12.0)	2,408 (0.7)	
Other (A, E, T)	33 (5.3)	2,738 (0.8)	
Acute change in consciousness, %	145 (23.3)	6595 (2.0)	<0.001
Vital signs at triage			
Systolic blood pressure, mean (SD), Mm Hg	122.2 (36.3)	136.2 (26.7)	<0.001
Heart rate, mean (SD), beats per minute	99.0 (28.6)	88.9 (19.1)	<0.001
Body temperature, mean (SD), °C	36.9 (1.3)	36.9 (0.8)	0.073
Respiratory rate, mean (SD), breaths per minute^a^	21.3 (4.9)	18.2 (2.2)	<0.001
Oxygen saturation, median (IQR), %	96 (92–98)	97 (96–99)	<0.001
Time to CPR, median (IQR), hr	7.0 (3.1–23.3)		
Length of ED stay, median (IQR), hr	8.7 (3.5–26.5)	2.8 (1.4–7.9)	<0.001
Discharge status, N (%)			<0.001
Discharge	0	252,998 (77.9)	
Admission	293 (47.0)	61,112 (18.8)	
Death	308 (49.4)	1,430 (0.4)	
Other^b^	22 (3.5)	9,329 (2.9)	

*IHCA*, in-hospital cardiac arrest; *SD*, standard deviation; *mm Hg*, millimeters of mercury; *GCS*, Glasgow Coma Scale; *A,E,T*, aphasia, tracheostomy, and endotracheal tube intubation; *GCS-A*, aphasia; *GCS-E*, endotracheal tube; *GCS-T*, tracheostomy

a Available in 546 IHCA and 307,767 non-IHCA patients.

b The 22 patients in the IHCA group left the hospital to die at home. The 9329 patients in the non-IHCA group were transferred to a nursing home, were discharged against medical advice, or left without being seen.

*IQR*, interquartile range; *CPR*, cardiopulmonary resuscitation; *ED*, emergency department; *hr*, hour.

**Table 2:** Multivariable analysis of factors associated with emergency department-based in-hospital cardiac arrest.

Variable	Adjusted Odds Ratio	95% Confidence Interval	*P*-value
Age (per 10-year increase)	**1.34**	1.25 – 1.42	<0.001
Female gender	1.19	0.97 – 1.47	0.099
Time of presentation			
7 AM to 2:59 PM	**1.30**	1.03 – 1.64	0.026
3 PM to 10:59 PM (reference)	1.00		
11 PM to 6:59 AM	**1.35**	1.003 – 1.81	0.047
Arrival by ambulance	**1.89**	1.46 – 2.45	<0.001
Transfer	**1.41**	1.04 – 1.89	0.025
Chief complaint			
Abdominal pain	1.22	0.76 – 1.95	0.414
Fever	0.85	0.55 – 1.3	0.451
Dyspnea	1.06	0.78 – 1.44	0.711
Dizziness	0.82	0.45 – 1.49	0.512
Chest pain	1.48	0.94 – 2.31	0.089
Other (reference)	1.00		
Triage level			
1	**2.48**	1.64 – 3.76	<0.001
2	**1.96**	1.51 – 2.54	<0.001
3 (reference)			
4	**0.33**	0.12 – 0.91	0.032
5	**0.30**	0.42 – 2.16	0.232
Pain score			
No pain (reference)			
Mild (1–3)	0.35	0.09 – 1.43	0.144
Moderate (4–6)	**0.63**	0.42 – 0.95	0.028
Severe (7–10)	**0.65**	0.46 – 0.94	0.021
GCS			
15 (reference)	1.00		
14	1.35	0.55 – 3.32	0.508
9–13	1.06	0.73 – 1.52	0.767
≤ 8	1.06	0.66 – 1.68	0.819
Other (A, E, T)	1.1	0.66 – 1.83	0.714
Vital signs at triage			
Systolic blood pressure < 90 mm Hg	**2.84**	2.05 – 3.91	<0.001
Heart rate			
< 60 beats per minute	**1.87**	1.15 – 3.02	0.011
60–90 (reference)	1.00		
> 90 beats per minute	**2.14**	1.68 – 2.73	<0.001
Body temperature			
< 36°C	**2.26**	1.69 – 3.04	<0.001
36–39 °C (reference)			
> 39°C	1.16	0.72 – 1.88	0.541
Respiratory rate ≥ 22 breaths per minute	**2.34**	1.76 – 3.11	<0.001
Oxygen saturation < 95%	**1.52**	1.18 – 1.96	0.001

Significant odds ratios are highlighted in bold.

*GCS*, Glasgow Coma Scale; *GCS-A*, aphasia; *GCS-E*, endotracheal tube; *GCS-T*, tracheostomy; *mm Hg*, millimeters of mercury.

**Table 3:** Condensed multivariable model of factors associated with emergency department-based in-hospital cardiac arrest.

Variable	Adjusted odds ratio	95% Confidence interval	*P*-value
Age ≥ 65 years	2.76	2.20 – 3.47	<0.001
Arrival by ambulance	2.11	1.66 – 2.67	<0.001
Systolic blood pressure < 90 mm Hg	4.03	2.97 – 5.46	<0.001
Heart rate			
< 60 beats per minute	2.16	1.33 – 3.50	0.002
60–90 (reference)	1.00		
> 90 beats per minute	2.26	1.86 – 2.99	<0.001
Body temperature < 36°C	2.61	1.95 – 3.49	<0.001
Respiratory rate ≥ 22 breaths per minute	3.18	2.46 – 4.12	<0.001
Oxygen saturation < 95%	1.94	1.52 – 2.48	<0.001
GCS < 15	1.57	1.19 – 2.07	0.001

*mm Hg*, millimeters of mercury; *GCS*, Glasgow Coma Scale.

**Table 4:** The items and scoring of the Emergency Department In-hospital Cardiac Arrest Score (EDICAS). The 8-item score ranges from 0 to 13.

Variable	Scoring		
	
	1	2	3
Age, year		≥ 65	
Arrival by ambulance	Yes		
Systolic blood pressure, mm Hg			<90
Heart rate, beats per min	<60 or > 90		
Body temperature, °C		<36	
Respiratory rate, breaths per minute		≥ 22	
Oxygen saturation, %	<95		
GCS < 15 or acute change in levels of consciousness	Yes		

*GCS*, Glasgow Coma Scale.

**Table 5:** Test characteristics of the Emergency Department In-hospital Cardiac Arrest Score (EDICAS).

Cut point	Risk category	Sensitivity, %	Specificity, %	PPV, %	NPV, %	LR+	LR−
≥ 1	Low	97	35	0.2	99.98	1.5	0.1
≥ 2		89	66	0.4	99.96	2.6	0.2

≥ 3	Medium	80	81	0.6	99.94	4.2	0.3
≥ 4		59	91	0.9	99.92	6.8	0.4
≥ 5		43	96	1.4	99.89	9.9	0.6

≥ 6	High	29	98	1.8	99.88	12.7	0.7
≥ 7		17	99	2.5	99.87	18.3	0.8
≥ 8		9	99	3.1	99.86	22.6	0.9
≥ 9		4	99	3.0	99.86	22.3	0.9
≥ 10		2	99	2.9	99.85	21.5	0.9
≥ 11		1	99	2.3	99.85	16.7	0.9
≥ 12		1	99	10.0	99.85	78.8	0.9

*PPV*, positive predictive value; *NPV*, negative predictive value; *LR+*, positive likelihood ratio; *LR*−, negative likelihood ratio.
