# Supplementary figures and images for: Development and Validation of a Novel Triage Tool for Predicting Cardiac Arrest in the Emergency Department
Source: West J Emerg Med. 2022 Feb 23;23(2):258–67. doi: 10.5811/westjem.2021.8.53063 (PMC8967450; doi:10.5811/westjem.2021.8.53063)

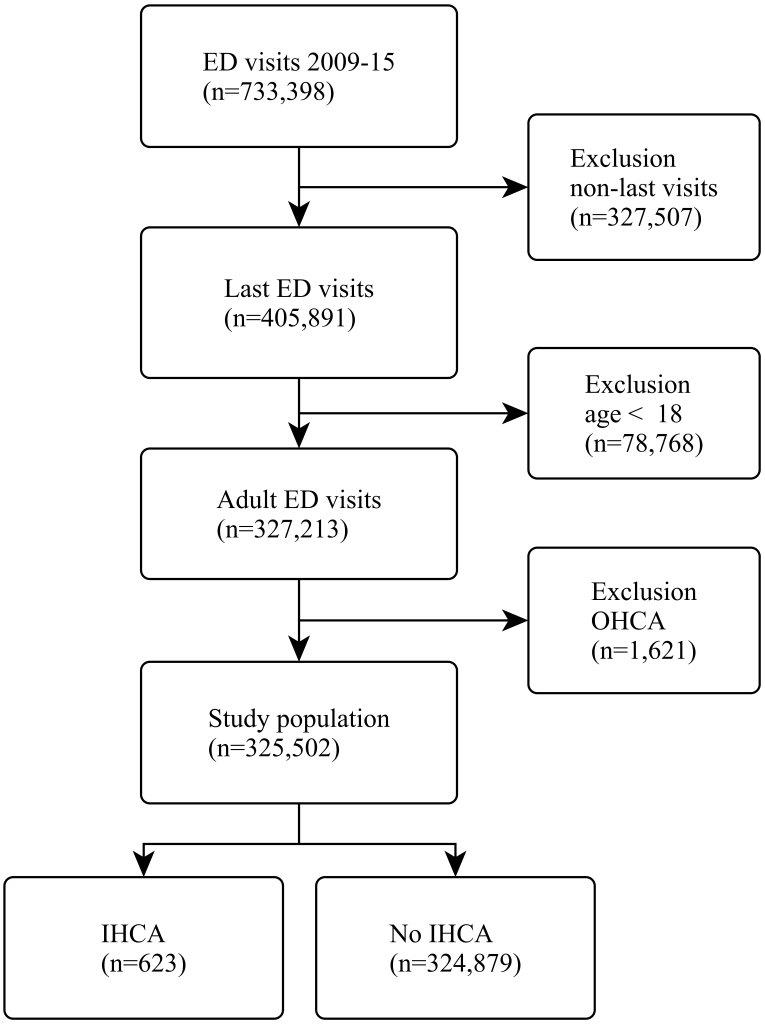

Supplement: Supplementary file 1 [file wjem-23-258-s001.tiff]

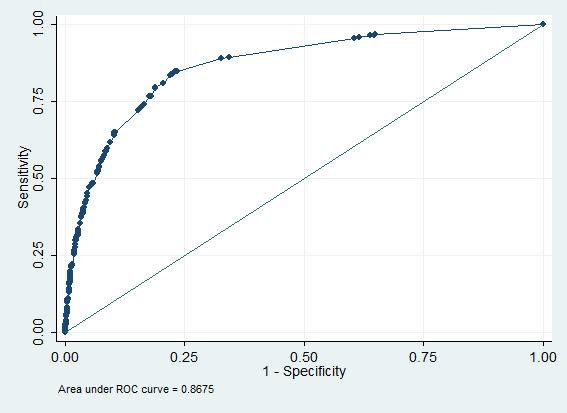

Supplement: Supplementary file 2 [file wjem-23-258-s002.tif]
