# Supplementary material for: Development and Validation of a Novel Triage Tool for Predicting Cardiac Arrest in the Emergency Department
Source: West J Emerg Med. 2022 Feb 23;23(2):258–67. doi: 10.5811/westjem.2021.8.53063 (PMC8967450; doi:10.5811/westjem.2021.8.53063)
Supplement: Supplementary file 3 [file wjem-23-258-s003.docx]

**Online Supplementary eTable 1**. Most common discharge diagnoses among the emergency department-based in-hospital cardiac arrests.

|  | Overall N=623 |
| --- | --- |
| Discharge diagnosis | n (%) |
| Pneumonia | 54 (8.7) |
| Chest pain | 31 (5.0) |
| Gastrointestinal hemorrhage | 30 (4.8) |
| Fever | 30 (4.8) |
| Cancer of head and neck | 21 (3.4) |
| Acute cerebrovascular disease | 21 (3.4) |
| Other lower respiratory disease | 21 (3.4) |
| Syncope | 21 (3.4) |
| Shock | 21 (3.4) |
| Renal failure | 20 (3.2) |

**Online Supplementary eTable 2.** Emergency Department In-hospital Cardiac Arrest Score (EDICAS); an alternative version with altered mental status.

| **Variable** | **Adjusted Odds Ratio** | **95% Confidence Interval** | **P value** |
| --- | --- | --- | --- |
| Age ≥ 65 years | 2.85 | 2.28 - 3.57 | <0.001 |
| Arrival by ambulance | 2.17 | 1.71 - 2.74 | <0.001 |
| Systolic blood pressure < 90 mmHg | 3.99 | 2.94 - 5.42 | <0.001 |
| Heart rate |  |  |  |
| < 60 beats per min | 2.13 | 1.31 - 3.45 | 0.002 |
| 60-90 (reference) | 1.00 |  |  |
| > 90 beats per min | 2.28 | 1.80 - 2.88 | <0.001 |
| Body temperature < 36°C | 2.58 | 1.92 - 3.45 | <0.001 |
| Respiratory rate ≥ 22 breaths per min | 3.30 | 2.56 - 4.25 | <0.001 |
| Oxygen saturation < 95% | 1.94 | 1.52 - 2.48 | <0.001 |
| Acute change in levels of consciousness | 1.72 | 1.24 - 2.37 | 0.001 |
